# Irregular rupture process of the 2022 Taitung, Taiwan, earthquake sequence

**DOI:** 10.1038/s41598-023-27384-y

**Published:** 2023-01-20

**Authors:** Yuji Yagi, Ryo Okuwaki, Bogdan Enescu, Junjie Lu

**Affiliations:** 1 Faculty of Life and Environmental Sciences, University of Tsukuba, Tennodai 1-1-1, Tsukuba, Ibaraki 305-8572 Japan; 2 Mountain Science Center, University of Tsukuba, Tennodai 1-1-1, Tsukuba, Ibaraki 305-8572 Japan; 3 Department of Geophysics, Graduate School of Science, Kyoto University, Kitashirakawa, Oiwake-cho, Sakyo-ku, Kyoto 606-8502 Japan; 4 National Institute for Earth Physics, Calugareni Str. 12, P.O. Box MG-2, 077125 Magurele-Bucharest, Ilfov Romania; 5 Graduate School of Science and Technology, University of Tsukuba, Tennodai 1-1-1, Tsukuba, Ibaraki 305-8572 Japan

**Keywords:** Seismology, Tectonics

## Abstract

In September 2022, two destructive earthquakes of moment magnitude (*M*_w_) 6.6 (foreshock) and 7.1 (mainshock) occurred in Taitung County, south-eastern Taiwan. To understand their complex rupture processes, we analysed these earthquakes using the Potency Density Tensor Inversion method, which can stably estimate the rupture propagation process, including fault geometry, without overfitting the data. The analyses revealed that the major rupture of the foreshock propagated towards shallow depth, in a south–southwest direction, following an initial rupture that propagated towards the deeper part of the fault. The mainshock, with its epicentre on the north–northeast side of that of the foreshock, consists of two distinct episodes. During the first episode (0–10 s), the initial rupture propagated north–northeast, through a deep path, followed by the main rupture that propagated bilaterally in a north–northeast and south–southwest direction. The second rupture episode (10–16 s) started near the hypocentre of the mainshock, and the rupture propagated towards the shallow side of the fault. The results suggest that the stress concentration from both the foreshock and mainshock’s first rupture episode may have caused the second rupture episode in the high fracture surface energy area between the foreshock and the first rupture episode of the mainshock. The irregular rupture process of the foreshock and mainshock may reflect the heterogeneity of stress and structure in the source region.

## Introduction

**Figure 1:**
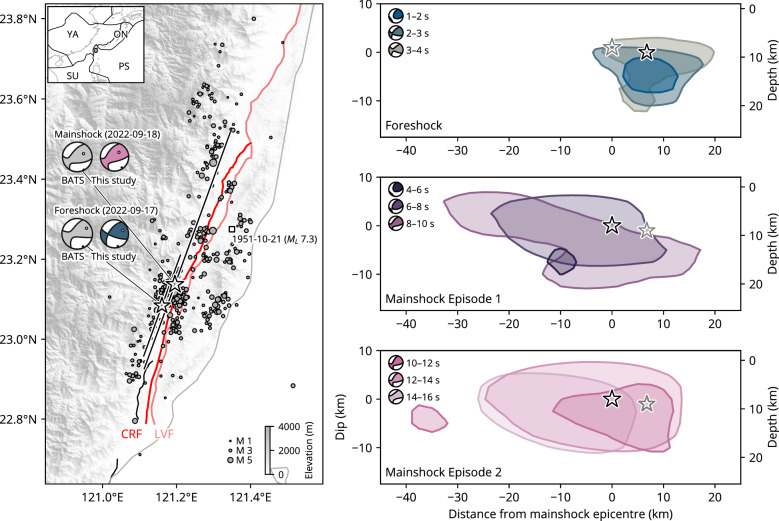
Overview of the 2022 Taitung earthquake sequence. The left panel shows the earthquake sequence (2022-09-17 to 2022-09-21) determined by the Central Weather Bureau (CWB) in Taiwan^1^. The size of the circle scales with its magnitude (see legend). The two stars show the largest foreshock and the mainshock epicentres, and black lines show top of the model plane used for our inversion. The square shows the epicentre of the *M*_*L*_ 7.3 earthquake on 21 October 1951^4^. The beachballs are the moment tensor solutions obtained by the Broadband Array in Taiwan for Seismology^3^ and this study. The dots on the beachball are the P and T axes. The light and dark red lines correspond to the Longitudinal Valley fault (LVF) and the Central Range fault (CRF)^7^. The topography is from the SRTMGL3^8^. The text denotes the plate name: YA; Yangtze, SU; Sunda, ON; Okinawa, and PS; Philippine Sea plates^9^. The right panels show the space distribution of the potency-rate density of the foreshock and the mainshock projected onto the mainshock model plane, viewed from the west-northwest. The contours in these figures are above 40% of maximum potency-rate density for each event: > 0.26 m/s for the foreshock and > 0.14 m/s for the mainshock.

On September 17, 2022 (UTC), a moment magnitude (*M*_W_) 6.6 earthquake occurred in Taitung County, Taiwan, which was about 16 h later followed by an *M*_W_ 7.1 earthquake (Fig. 1). We refer to the *M*_W_ 6.5 earthquake as the largest foreshock of the 2022 MW 7.1 Taitung mainshock. The locations of foreshocks and aftershocks of the 2022 Taitung earthquake sequence have been determined by the Central Weather Bureau (CWB) in Taiwan, and they are distributed along a direction trending NNE-SSW^1^ (Fig. 1). The centroid moment tensor (CMT) solutions of the Broadband Array in Taiwan for Seismology (BATS)^2,3^, indicate that both the largest foreshock and the mainshock have a strike-slip fault mechanism with a reverse component; the mainshock has a more predominant reverse component than the largest foreshock (Fig. 1). The nodal plane of the CMT solution, whose strike coincides with the direction of the aftershock distribution, shows a fault plane dipping to the west for both the largest foreshock and the mainshock. Two parallel faults, the east-dipping Longitudinal Valley fault (LVF) and the west-dipping Central Range fault (CRF), extend along a north-northeast to south-southwest direction^4–6^, overlapping the aftershock area (Fig. 1). A local magnitude (*M*_L_) 7.3 earthquake along the LVF fault occurred in this area in 1951^4^.

There are two approaches to analyse earthquakes occurring in complex fault systems. One approach is to construct a detailed and complex fault model, which can explain near source fault displacements^10–13^. The other approach is to construct an overall seismic source model using a Potency Density Tensor Inversion (PDTI) method that does not require the definition of a fault plane^14^. The former can construct a detailed earthquake rupture model, however the Green's function is sensitive to the details of the assumed fault plane, so the solution obtained would also be sensitive to the fault plane characteristics^15,16^. On the other hand, the later approach can stably estimate the spatio-temporal distribution of the potency-rate density tensor, including information on slip vectors and fault geometry, although it is difficult to identify the ruptured fault plane^17–19^. The PDTI introduces the Akaike’s Bayesian Information Criterion (ABIC), which prevents overfitting of the data, so that the performed analyses are stable even in case of using seismic source models with a large number of model parameters^17,20–22^.

In this study, the PDTI method is applied to the tele-seismic P-waves of the largest foreshock and the mainshock of the 2022 Taiwan earthquake sequence to clarify their seismic source processes. We observe, for the mainshock, a delayed main rupture that initiated around the hypocentre at 10–16 s from the start of the rupture, which predominantly occurs in the slip deficit area sandwiched by the largest foreshock and the initial mainshock rupture areas. The two mainshock episodes are not smoothly connected and, therefore, can be referred to as a doublet earthquake, occurring within a short time interval.

## Method and data

We use the PDTI method, which analyses the seismic source process assuming a model plane rather than a fault plane. This method projects the faulting slip on multiple fault planes as a potency density tensor by setting the five-component basis double-couple components^23^ along the model plane. Considering that tele-seismic body waves are sensitive to changes in the focal mechanism, the PDTI builds a source process model including variations in the focal mechanism, thereby reducing the effects of modelling errors due to differences between the model plane and the true fault plane^14^. In this study, we used the latest version of the PDTI method, which introduces time-adaptive smoothing^24^.

Tele-seismic P-waves, downloaded from the Incorporated Research Institutions for Seismology Data Management Center (IRIS-DMC), were used in the analyses. The data at 42 seismic stations were used for the largest foreshock analysis and 41 for the mainshock analysis; there were 38 stations common for both analyses (Figs. S3, S4). The observed waveforms were converted to velocity waveforms by removing the instrument response and then decimating the signal by using a 0.8-s sampling. The Green’s function was calculated using the code of Kikuchi & Kanamori^23^ assuming CRUST1.0^25^ for the one-dimensional structure of the source region (Table S1). The uncertainty in the Green's function was introduced into the data covariance matrix following Yagi and Fukahata^26^. The effect of the Earth’s 3-D velocity structure was reduced by manually picking P waves. For the structure of the source region, we also performed the PDTI analysis using two alternative structures (Fig. S1).

The strike and dip of the model plane were set to 200° and 90° for both the foreshock and the mainshock (Fig. 1). With reference to the foreshock and aftershock distributions, the model area was set to 30 km × 26 km for the foreshock and 70 km × 27 km for the mainshock, with a spatial node interval of 2.5 km. The initial rupture point (latitude, longitude, depth) of the largest foreshock and the mainshock were set at (23.084° N, 121.161° E, 9 km) and (23.137° N, 121.196° E, 8 km), respectively, based on the ones determined by the CWB^1^. To the best of our knowledge, there have been no reports of significant surface ruptures, so we constrain the potency-rate density to be zero at 2.5 km above the uppermost spatial node. All the basis double-couples were rotated so that one component of the basis double-couple corresponded to the best-fit double-couple of the Global Centroid Moment Tensor (GCMT) solution^27,28^. The maximum rupture-front velocity was set to 4.0 km/s, so that our seismic source model with rupture-front velocities exceeding the S-wave can also be represented. The potency-rate density tensor function for each spatial node was assumed to be a linear B-spline function with 0.8 s intervals, assuming a total duration of 6.4 s and 25.6 s for the largest foreshock and the mainshock, respectively.

## Results

**Figure 2:**
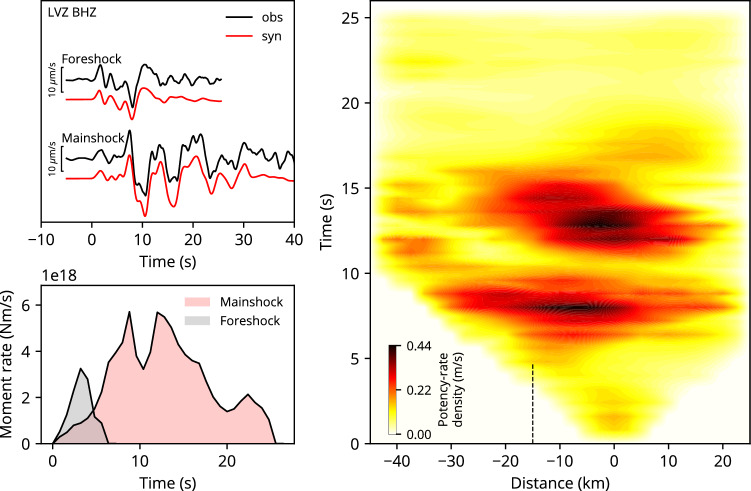
The left-top panel shows waveform fitting for the largest foreshock and the mainshock at the selected station (LVZ). The left-bottom panel shows the moment-rate functions. The right panel shows potency-rate density evolution projected along-strike (200° azimuth) distance from the mainshock epicentre. The black dashed line indicates the location where the rupture apparently stagnates, approximately 15 km north-northeast of the mainshock epicentre.

The epicentre of the largest foreshock is located approximately 8 km southwest of the mainshock epicentre. The rupture started propagating from the hypocentre to the deeper part of the fault. After this initial rupture episode, the main rupture propagated towards the south-southwest, shallower part of the fault, 2 s after the initial break and was almost completed after 5 s (Figs. 1, S2). The moment-rate function peaks at 3.2 s after the initial break (Fig. 2). The obtained seismic moment is 8.9 × 10^18^ Nm (*M*_W_ 6.6), which agrees well with the GCMT solution of 8.7 × 10^18^ Nm (*M*_W_ 6.6). Our moment tensor solution indicates strike-slip faulting with a minor reverse component, with a northwest-southeast oriented P-axis and northeast-southwest oriented T-axis (Fig. 1). The focal mechanism changes through time, as the strike-slip component becomes dominant (Figs. 1, S2).

The hypocentre of the mainshock is located at the north–northeast edge of the main rupture zone of the largest foreshock (Fig. 1). The obtained seismic moment is 6.1 × 10^19^ Nm (*M*_W_ 7.1), which is larger than the GCMT solution of 3.9 × 10^19^ Nm (*M*_W_ 7.0). Our moment tensor solution shows that the earthquake is a strike-slip with reverse component, with a northwest–southeast P-axis and a northeast-southwest T-axis (Fig. 1). The T-axis plunge of the mainshock is larger than that of the largest foreshock, and the reverse component is more dominant in the mainshock. The moment-rate function has two large peaks at 8.8 and 12 s after the initial break (Fig. 2). Corresponding to these two peaks, rupture evolution can be divided into two episodes, one from the start of rupture to 10 s and the other from 10 to 20 s.

During the first episode, after the initial rupture propagated in the north-northeast direction for the first 4 s, the main rupture propagated bilaterally in a north-northeast and south-southwest direction (Fig. 1). Propagation of the north to north-eastward rupture stalled between 5 and 7 s around 15 km north-northeast of the epicentre and then rapidly accelerated and ceased at around 10 s (Figs. 2, S2). During the second episode, from about 10 s after the initial break, rupture began below the mainshock hypocentre, propagated towards shallower depths, and was almost completed at 16 s (Fig. 2). No significant sub-event can be detected after 16 s. The first episode has predominantly a reverse component compared to the second episode (Fig. 1).

The synthetic waveforms for both the largest foreshock and the mainshock explain well the observed waveforms, including not only the data points used for inversion but also those that were not used (Figs. 2, S3, S4).

## Discussion and conclusions

For both the largest foreshock and the mainshock, our PDTI results revealed that the rupture area did not expand monotonically from the hypocentre, but rather contained a complicated, cascading rupture network. During the largest foreshock, the rupture propagated once towards the deeper region of the fault, then it advanced towards the south-southwest and shallow fault region. The mainshock rupture can be divided into two episodes. During the first episode, the rupture propagated unilaterally to the north–northeast and then propagated bilaterally, starting around 10 km north–northeast of the epicentre, while in the second episode, rupture started below the mainshock hypocentre and propagated towards the shallow area. It should be noted that it is difficult to discuss in detail the distribution of potency density tensor near the surface from the tele-seismic P-waves because the amplitudes of the Green's functions corresponding to near-surface nodes are smaller than those of deeper nodes.

Since there is some possibility that the second episode observed during the mainshock rupture may have been artificially generated by inappropriate structure assumptions^29^, we performed an inversion analysis using two alternative structures and confirmed that the delayed rupture around the mainshock hypocentre corresponding to the second episode was robustly obtained (Fig. S1). The mainshock can be interpreted as a doublet earthquake, occurring within a short time interval, as the two episodes of the mainshock are not smoothly connected (Fig. S1). We also analysed the mainshock with a model plane dipping at 60° that is similar to the GCMT solution and confirmed that such features can be reproduced (Fig. S1). On the other hand, after 14 s from the initial break, the features of the potency density distribution tend to depend on the assumed structural model. The direction of rupture propagation in the second episode seems to be structure-dependent, as we could identify two different patterns: rupture propagating in a north-northeast direction when using the CRUST 1.0 and 2.0 models^25^ (Fig. S1a,b) and rupture stagnating near the epicentre when using the semi-infinite model (Fig. S1c). Therefore, it is difficult to discuss the horizontal propagation of the second episode using only the PDTI results.

Assuming the rupture occurred on the west-dipping fault plane among the two nodal planes obtained from the focal mechanism of both the largest foreshock and the mainshock, it can be inferred that the rupture propagated along the west-dipping fault plane during both the largest foreshock and the mainshock. The strike and dip of this west-trending fault plane are 213° and 58°, respectively, for the foreshock, and 201° and 47°, respectively, for the mainshock, which are consistent with the fault geometry of the Central Mountain Tectonic Fault^30,31^. On the other hand, our PDTI with tele-seismic P waves cannot detect a sub-event with a focal mechanism corresponding to the east-dipping Longitudinal Valley Fault, where both aseismic and seismic slip have been observed^5,32^.

Comparison of the largest foreshock and the first episode of the mainshock shows that the major rupture areas are spatially complementary, with the former having a predominant strike-slip component compared to the latter (Fig. 1). The second episode of the mainshock, on the other hand, begins near the middle of the rupture zone of the largest foreshock and the first episode of the mainshock, and appears to partly overlap the rupture zone of the largest foreshock and the first episode (Fig. 1). It should be noted, however, that the PDTI can project multiple fault slips onto the assumed model plane, so it is not possible to determine specifically if the same region has ruptured.

Although proposing a dynamic rupture model is beyond the scope of this study, it is interesting to discuss how such a complex rupture sequence could have occurred. There are at least two possible models to explain the complex rupture sequence of the 2022 Taitung earthquakes. The hierarchical asperity model^33,34^ could be one such model; alternatively, one can assume some (possibly unknown) parallel faults that are connected deep underground (deep-rooted fault model). In the case of the hierarchical asperity model, the complex rupture sequence can be explained by considering that the “strongest” patch (high fracture surface energy area) is distributed between the rupture areas of the largest foreshock and the first episode of the mainshock. Based on the hierarchy of asperities, the strongest patch can inhibit or promote rupture growth, depending on the degree of shear stress concentration. During the largest foreshock and the first mainshock episode, there was insufficient stress concentration to rupture the strongest patch of the fault. On the other hand, fault slip on both sides of the strong patch increased the shear stress enough to rupture this area during the second rupture episode. According to the deep-rooted fault model, the rupture propagates on one of the two parallel faults in the first episode, and, in the second episode, the rupture moves upward from deep underground (where the two faults connect) to the other fault. A similar phenomenon has been observed for the 2008 Wenchuan, China earthquake^35–37^. However, the deep-rooted model may be unlikely since the south-southwest rupture of the first episode is not smoothly connected to the rupture of the second episode (Fig. 2) and the two parallel fault planes cannot be clearly identified from the aftershock distribution (Fig. S5).

The PDTI results based on tele-seismically recorded body waves reveal a complex series of seismic source processes for the 2022 Taitung earthquake sequence. Specifically, we resolved the irregular rupture processes associated with a change of rupture propagation direction. Such “flips” of rupture direction have been confirmed for other earthquakes by the back-projection method and high-degree-of-freedom inversions^38–45^. The irregular rupture propagation in the source region of the 2022 Taitung earthquakes, including delayed rupture near the mainshock hypocentre and flipping of rupture propagation directions, may reflect apparent strength and stress heterogeneity along the fault plane related to the complex fault geometry.

It should be worthwhile to point out that the strong ground motions of north–south component observed near the epicentre, which area nearly parallel to the fault plane, have some interesting features^1^ (Fig. S6). A large amplitude signal was observed about 15 s from the origin time at the TW.G023 station, located about 10 km south-southwest of the epicentre and close to the second rupture episode area, while a large amplitude signal was observed about 9 s after the origin time at the TW.F042 station located about 10 km north-northeast of the epicentre. In addition to this, the strong ground motion at station TW.G021, which is closer to the epicentre, tends to have a longer duration than at the other stations and appears to contain signals associated with two major sub-events. Although these features of the strong ground motion records are consistent with the results of this study, it may be difficult to conclude that these records support our results, as the Green's function near faults is sensitive to slight changes in the geometry of the fault plane.

To understand the complex rupture propagation process of the 2022 Taitung, Taiwan, earthquakes, it will be important to perform in a future study a detailed analysis of near-source data, with the proper fault model. The overall seismic source model obtained by the PDTI method, whose solution is less distorted by the assumed model plane, should promote more objective and detailed studies of the seismic source process of the 2022 Taitung triplet earthquake sequence.

## Supplementary Information

Supplementary Information.

## Data Availability

All seismic data were downloaded through the IRIS Wilber 3 system (https://ds.iris.edu/wilber3/), including the following seismic networks: the GEOSCOPE (G; http://geoscope.ipgp.fr/networks/detail/G/); the GEOFON (GE; https://geofon.gfz-potsdam.de/doi/network/GE); the IRIS/IDA Seismic Network (II; https://www.fdsn.org/networks/detail/II/); the Global Seismograph Network (IU; https://www.fdsn.org/networks/detail/IU/). The centroid moment tensor solutions are obtained from the GCMT catalog (https://www.globalcmt.org/CMTsearch.html) and AutoBATS CMT catalog (https://tecdc.earth.sinica.edu.tw/FM/AutoBATS/). The CRUST1.0 and CRUST2.0 structural velocity models are available through the websites https://igppweb.ucsd.edu/~gabi/crust1.html and https://igppweb.ucsd.edu/~gabi/crust2.html, respectively. The global database of the major active faults from Styron and Pagani (2020) is available at https://github.com/GEMScienceTools/gem-global-active-faults. The hypocentre information and strong ground motion data are available through the Geophysical Database Management System (GDMS) in CWB (https://gdmsn.cwb.gov.tw).
