# Supplementary material for: Irregular rupture process of the 2022 Taitung, Taiwan, earthquake sequence
Source: Sci Rep. 2023 Jan 20;13:1107. doi: 10.1038/s41598-023-27384-y (PMC9859779; doi:10.1038/s41598-023-27384-y)
Supplement: Supplementary file 1 — Supplementary Information. [file 41598_2023_27384_MOESM1_ESM.pdf]

Irregular Rupture Process of the 2022 Taitung, Taiwan, Earthquake Sequence

Yuji Yagi[1,2], Ryo Okuwaki [1,2], Bogdan Enescu[3,4], Junjie Lu[5]
[1]Faculty of Life and Environmental Sciences, University of Tsukuba, Tennodai 1-1-1, Tsukuba, Ibaraki 305–8572, Japan
[2]Mountain Science Center, University of Tsukuba, Tennodai 1-1-1, Tsukuba, Ibaraki 305–8572, Japan
[3]Department of Geophysics, Graduate School of Science, Kyoto University, Kitashirakawa, Oiwake-cho, Sakyo-ku, Kyoto 606-8502, Japan
[4]National Institute for Earth Physics, Calugareni str. 12, P.O. Box MG-2, 077125, Magurele-Bucharest, Ilfov, Romania
[5]Graduate School of Science and Technology, University of Tsukuba, Tennodai 1-1-1, Tsukuba, Ibaraki 305–8572, Japan

* Corresponding author (yagi-y@geol.tsukuba.ac.jp)

Contents

Table S1: A structure model (CRUST1.0) used for calculating Green's functions
Table S2: An alternative structure model (CRUST2.0) used for calculating Green's functions
Table S3: An alternative structure model (semi-infinite model) used for calculating Green's functions
Figure S1: A model using alternative velocity structure
Figure S2: Snapshots of models for the mainshock and foreshock
Figure S3: Waveform fits of the largest foreshock model
Figure S4: Waveform fits of the mainshock model
Figure S5: Example traces of near-field strong motion records of the mainshock

Table S1: Structure from CRUST1.0[1] used for calculating Green's functions

V_P (km/s)	V_S (km/s)	Density (10^3 kg/m^3)	Thickness (km)
5.80	3.40	2.63	14.47
6.30	3.62	2.74	12.86
6.90	3.94	2.92	12.85
7.75	4.32	3.20	- (Moho)

Table S2: An alternative structure from CRUST2.0[1] used for calculating Green's functions

V_P (km/s)	V_S (km/s)	Density (10^3 kg/m^3)	Thickness (km)
6.5	3.5	2.7	15.0
6.6	3.7	2.9	9.0
7.2	4.0	3.05	11.0
8.0	4.6	3.3	- (Moho)

Table S3: An alternative structure (semi-infinite model) used for calculating Green's functions

V_P (km/s)	V_S (km/s)	Density (10^3 kg/m^3)	Thickness (km)
5.80	3.40	2.63	-

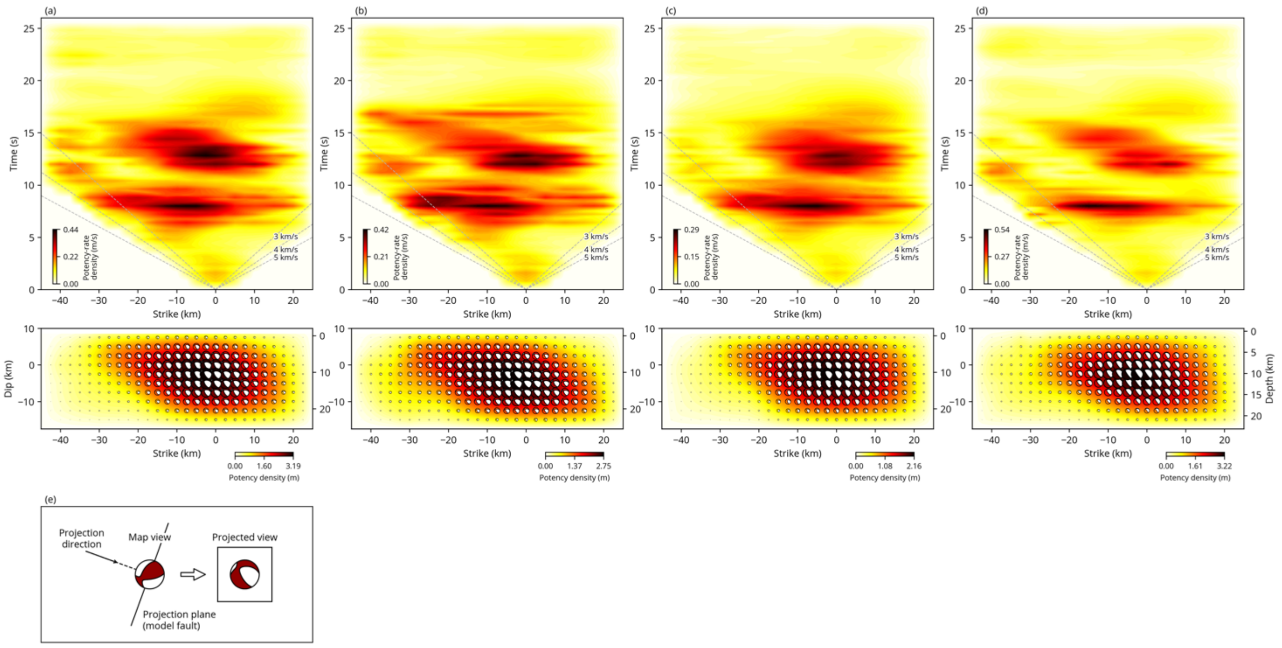

Figure S1: A comparison of potency-rate density evolution and total potency tensor distribution using (a) CRUST1.0 model (Table S1), (b) CRUST2.0 model (Table S2), (c) a semi-infinite structure model (Table S3) and (d) CRUST1.0 model and the model-plane-dip of 60°. The top panel of each case shows potency-rate density evolution projected along-strike distance from the mainshock epicenter. The bottom panel of each case shows total potency tensor distribution projected onto the mainshock model plane. The beachball is the moment tensor solution viewed from the west-northwest. (e) Schematic diagram of the beachball projection. Both pre- and post-rotation moment tensor solution are shown in the lower hemisphere projection.

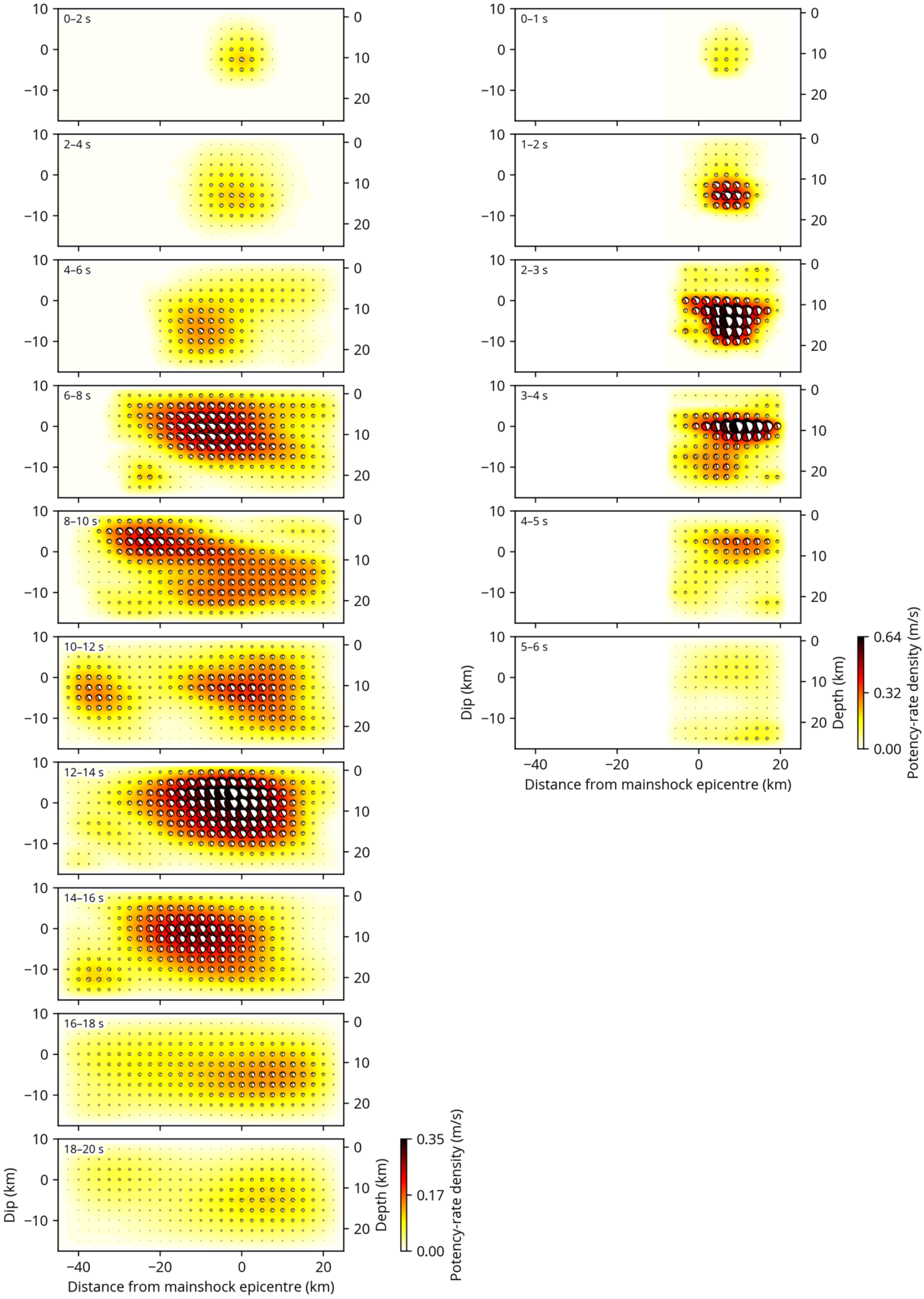

Figure S2: Snapshots of models for the mainshock (left panels) and largest foreshock (right panels). The distance (abscissa) is measured on the projected line along the strike direction of the mainshock model fault. The way of plotting beachballs is the same as that of Fig. S1.

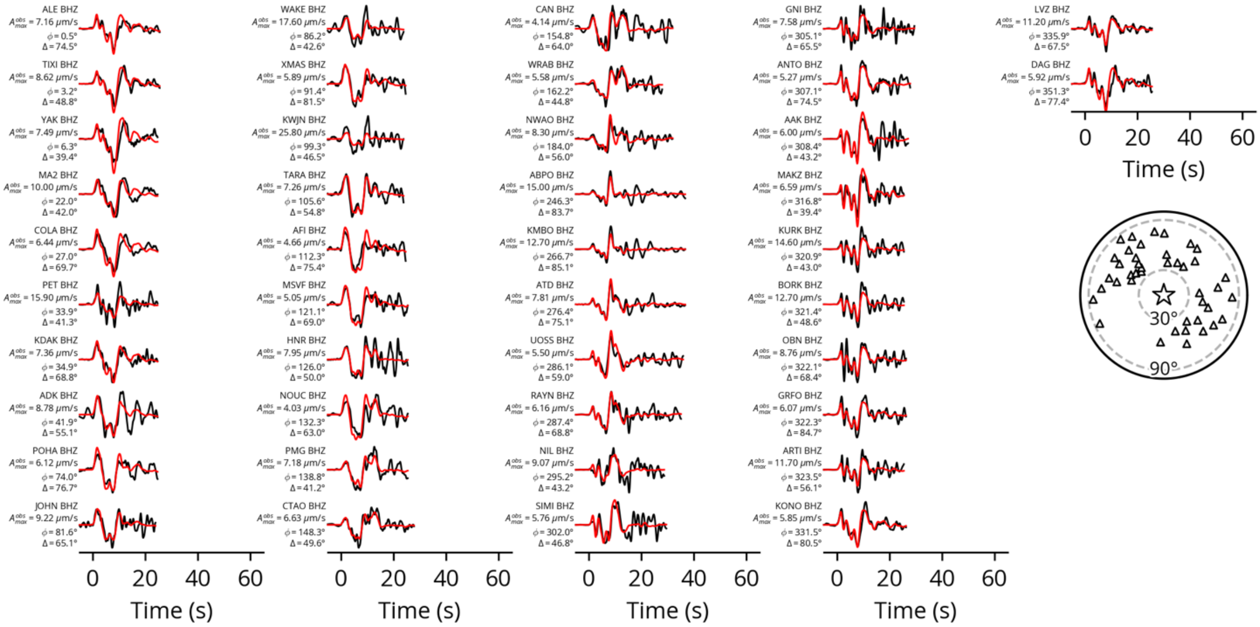

Figure S3: Waveform fits of the largest foreshock model. The black and red traces are the observed and synthetic waveforms, respectively. The station code, channel, maximum amplitude of the observed data, station azimuth (ϕ), and epicentral distance (Δ) are shown on left of each panel. The normalized residual (sum of squares of the difference between observed and theoretical data normalized by the sum of squares of the observed data) of the data used in the inversion analysis is 0.36. The right-bottom panel shows the station distribution (triangle). The star denotes the epicentre. The dashed lines are the reference epicentral distances.

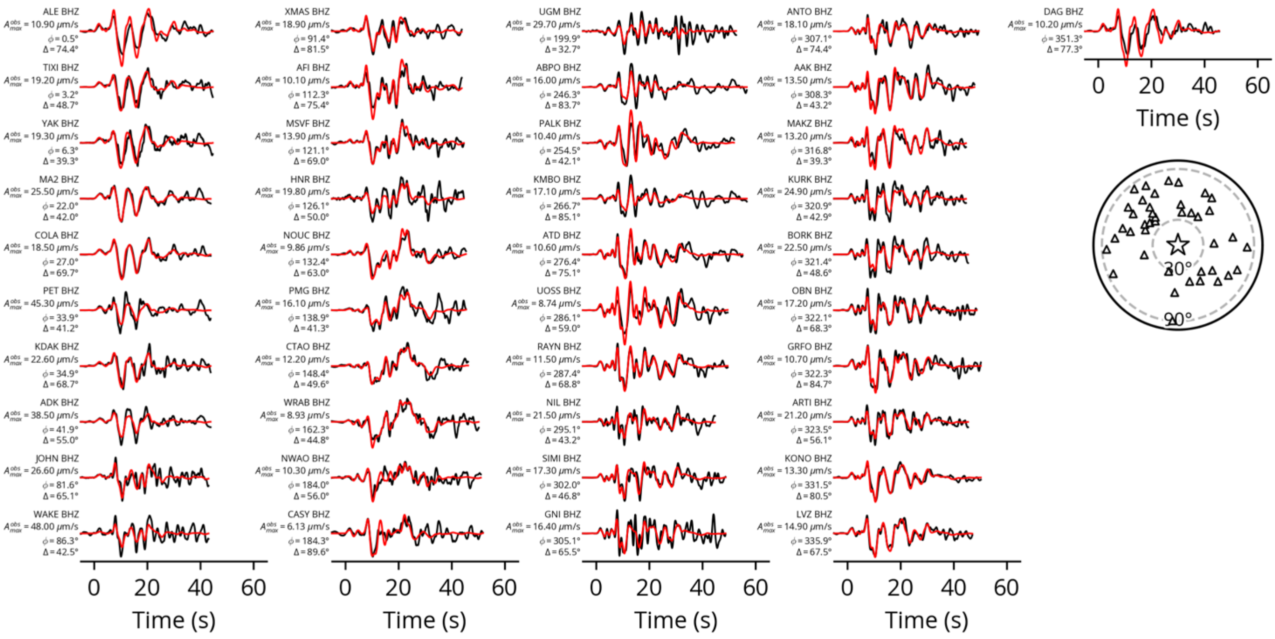

Figure S4: Waveform fits of our preferred mainshock model. The black and red traces are the observed and synthetic waveforms, respectively. The station code, channel, maximum amplitude of the observed data, station azimuth (ϕ), and epicentral distance (Δ) are shown on left of each panel. The normalized residual of the data used in the inversion analysis is 0.31. The right-bottom panel shows the station distribution (triangle). The star denotes the epicentre. The dashed lines are the reference epicentral distances.

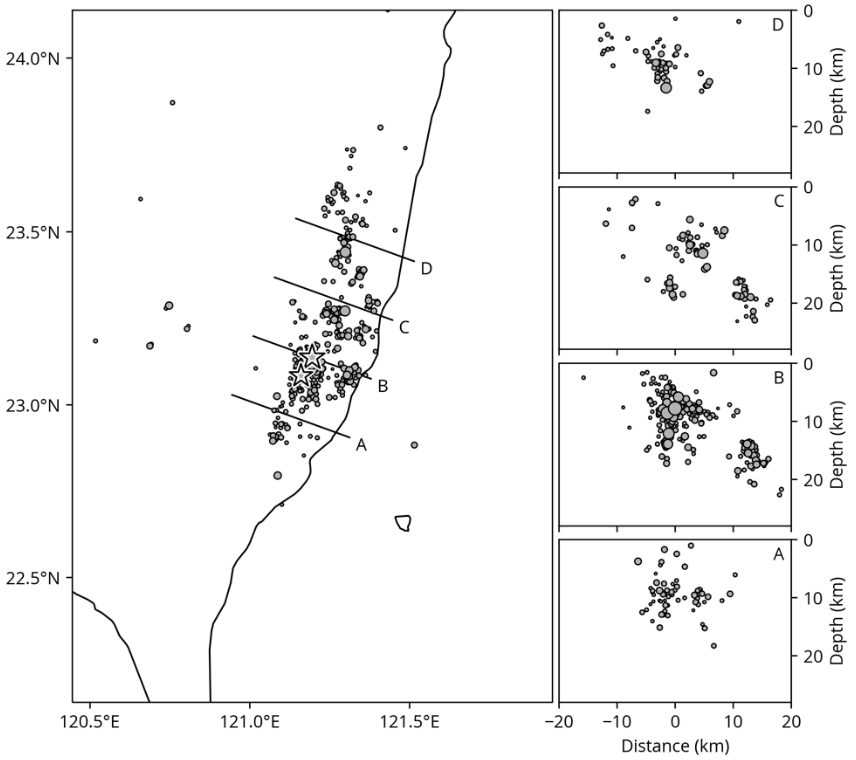

Figure S5: The earthquake sequence (2022-09-17 to 2022-09-21) determined by CWB Taiwan (2012)[2]. The right panels show the cross sections of the aftershock distribution along the A, B, C and D lines in the left panel, respectively. The stars in the left panel represent the foreshock and mainshock epicentres[2].

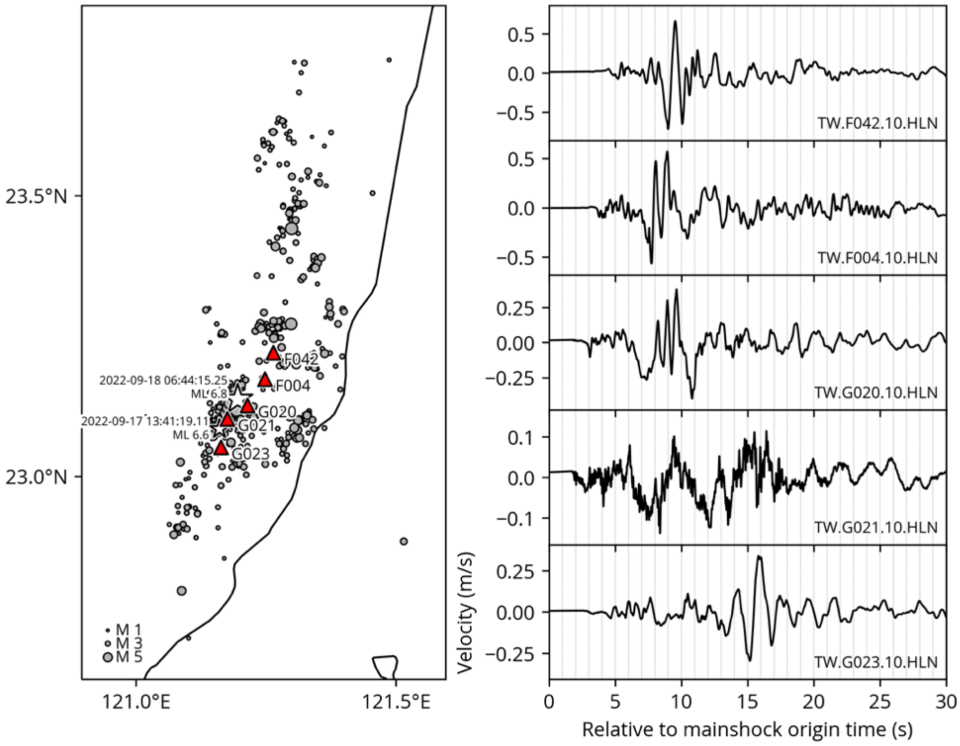

Figure S6: Example traces of near-field strong motion records of the mainshock from CWB Taiwan (2012)[2]. The stars (foreshock and mainshock) and the dots (earthquake sequence during 2022-09-17 and 2022-09-21) are from CWB Taiwan (2012)[2]. The location of each station is shown on a map (triangle). The black lines are the coastlines. The station network, code and channel are denoted on bottom-right of each trace panel.

References

1. Laske, G., Masters, T. G., Ma, Z., & Pasyanos, M. (2013). Update on CRUST1.0 - A 1-degree Global Model of Earth's Crust. EGU Gen. Assem. **15**, 2658 (2013)
2. Central Weather Bureau (CWB, Taiwan). (2012). Central Weather Bureau Seismographic Network [Data set]. International Federation of Digital Seismograph Networks. https://doi.org/10.7914/SN/T5
